# Supplementary material for: The hidden/ hard to reach men who have sex with men (MSM)—Results from the qualitative study in India
Source: PLoS One. 2026 Jan 23;21(1):e0340282. doi: 10.1371/journal.pone.0340282 (PMC12829927; doi:10.1371/journal.pone.0340282)
Supplement: Attachment — Submitted filename: Response_to_Reviewer_Comments.docx [file pone.0340282.s002.docx]

**The line numbers are corresponding to the manuscript with track changes.**

| **Sr no** | **HRMSM comments** | **Response** | **Page and line number** |
| --- | --- | --- | --- |
| **Before we proceed with your manuscript, please address the following prompts:** | | | |
| 1 | a) If there are ethical or legal restrictions on sharing a de-identified data set, please explain them in detail (e.g., data contain potentially identifying or sensitive patient information, data are owned by a third-party organization, etc.) and who has imposed them (e.g., a Research Ethics Committee or Institutional Review Board, etc.). Please also provide contact information for a data access committee, ethics committee, or other institutional body to which data requests may be sent. | Thank you very much we have addressed this issue in the manuscript. | Data Availability Statement, Page 56, Line no. 1221-1225 |
| 2 | b) If there are no restrictions, please upload the minimal anonymized data set necessary to replicate your study findings to a stable, public repository and provide us with the relevant URLs, DOIs, or accession numbers. Please see <http://www.bmj.com/content/340/bmj.c181>.long for guidelines on how to de-identify and prepare clinical data for publication. For a list of recommended repositories, please see <https://journals.plos.org/plosone/s/recommended-repositories>. You also have the option of uploading the data as Supporting Information files, but we would recommend depositing data directly to a data repository if possible. | There are restrictions on publically sharing this study data due to its sensitive nature. However data is available at center repository |  |
| 3 | Please update your Data Availability statement in the submission form accordingly. | The data availability statement is been updated |  |
| 4 | For studies involving third-party data, we encourage authors to share any data specific to their analyses that they can legally distribute. PLOS recognizes, however, that authors may be using third-party data they do not have the rights to share. When third-party data cannot be publicly shared, authors must provide all information necessary for interested researchers to apply to gain access to the data. (<https://journals.plos.org/plosone/s/data-availability#loc-acceptable-data-access-restrictions>) | The contact details for the interested researcher to gain access to the data has been provided. (Refer to point no. 1) |  |
| 5 | For any third-party data that the authors cannot legally distribute, they should include the following information in their Data Availability Statement upon submission:  1) A description of the data set and the third-party source  2) If applicable, verification of permission to use the data set  3) Confirmation of whether the authors received any special privileges in accessing the data that other researchers would not have  4) All necessary contact information others would need to apply to gain access to the data  5. If the reviewer comments include a recommendation to cite specific previously published works, please review and evaluate these publications to determine whether they are relevant and should be cited. There is no requirement to cite these works unless the editor has indicated otherwise. | 1. Description of data set which is direct experiences of hidden men who have sex with men has been added (refer to point 4). There is no third party source.  2. Use of data set is freely available. The researcher needs to send a request (refer point 1 or 4)  3. No special privileges were received by authors  4. A sound research question will be needed to apply to gain access to the data  5.None of the reviewers has recommended to cite any published work |  |
| **Additional Editor Comments:** | | | |
| 6 | Please review and address the concerns raised by the reviewers, particularly reviewer 2, regarding the methodological approach and analysis reported.  Clarity on how this paper relates to the already published study, whether previous results have been previously reported, and how those results differ from this paper is needed. | Thank you we have mentioned about the differences between the previous article published and this current paper. | Discussion, page 42, line no.  901-906 |
| **Review Comments to the Author**  Please use the space provided to explain your answers to the questions above. You may also include additional comments for the author, including concerns about dual publication, research ethics, or publication ethics. (Please upload your review as an attachment if it exceeds 20,000 characters) | | | |
| 7 | Reviewer #1: The data gathered through the study is very relevant and useful for potential improvement in the implementation of programs targeting MSM, not only in India but also in other countries. Overall, I felt that the article is very long which to some extent makes sense given the quotes from participants and the very diverse categories that were included. Nevertheless, the focus can get lost given the amount of categories and information. If possible, I suggest making it more concrete but I don't think this is an obstacle to accepting the paper. | Sincere thanks. We have tried to reduce the length of the manuscript to the best of our endeavors. |  |
| 8 | A table showing the characteristics of participants would be useful to get a better sense of who is represented in the study. Also, the text in the quotes from participants may be difficult to understand for some people who are not used to the literal transcript of interviews, I suggest to simplify it. | Thank you a table has been added as suggested. The quotes have been revised to make it simpler and comprehensive although still keeping it verbatim. | Page 65-67. Table 1 and 2  Quotes  Page 11, line no. 223-224, Page 12, line no. 229  Page 13, line no. 262  Page 14, line no. 280-282  Page 15, line no. 314  Page 16, line no. 329  Page 18, line no. 374  Page 20, line no. 389  Page 21, line no. 414-416, 420  Page 21-22, line no. 431, -438  Page 24, line no. 482,  491, 494, 495, 497, 501, 502  Page 25, line no. 506, 511  Page 28, line no. 575, 579, 580, 586  Page 30, line no. 623  Page 31, line no. 661  Page 32, line no. 685-686  Page 33, line no. 693, 709  Page 34, line no. 720, 726,733,  Page 36, line no. 766, 780  Page 38, line no. 824  Page 39, line no. 831  Page 39, line no. 845,847 |
| **Reviewer #2: GENERAL COMMENTS ON THE ARTICLE** | | | |
| 9 | The article presents original research data and addresses a crucial and highly relevant topic for HIV prevention in India, which may be of great use in the global fight against HIV. The empirical evidence included in the manuscript is extensive, and its results are robust. However, there are both technical and conceptual elements that would benefit from further review. | Thank you for the encouraging feedback |  |
| **ON TIMELINESS** | | | |
| 10 | Relevance: The authors could initially elaborate on the relevance of their data, considering that these qualitative data were collected six years ago (2019), before the COVID-19 pandemic, which significantly altered health services worldwide. It would be advisable for the authors to explain why the data remain relevant today. | As suggested the relevance of the data in today context has been added in the revised manuscript. | Limitation, page 51-52,  line no. 1134-1140 |
| 11 | **ON THE OBJECTIVE OF THE ARTICLE** |  |  |
|  | In the article it is stated: “This study was conducted among MSM and other stakeholders to understand the population which is hidden, their issues and their expectations on how to reach them comfortably; without any threat.”  **Comment:** In this sentence you outline the study’s objective; however, I believe that the article’s objective could be more specific. It would be worthwhile to review and adapt this objective so that it aligns more directly with the manuscript’s findings. | As suggested we have revised the objectives as follows. "This paper is a synthesis of evidence generated on prevalent psychosocial issues among hidden MSM, risks experienced, their networking, drivers of HIV risk, and mental health issues." | Introduction, page 6,  Line no. 102-104 |
|  | ANSWERING THE QUESTION: IS THE MANUSCRIPT WELL ORGANIZED AND WRITTEN CLEARLY ENOUGH TO BE ACCESSIBLE TO NON-SPECIALISTS?  No | We have tried to reorganize the revised manuscript while making it simpler for comprehension of non-specialist. |  |
| 12 | LENGTH OF THE DOCUMENT AND LACK OF BALANCE IN SECTIONS  Comment: The article is unnecessarily long, with 11,000 words. The Introduction has 1.5 pages (564 words), the Methods 2 pages (530 words), the Results 23 pages (6,902 words), and the Discussion 6 pages (2,624 words).  The Results section reads more like a research report than a journal article.  It is recommended to reduce the length, particularly of the Results. This could be achieved by selecting key testimonies, since many can serve multiple analytical points. Another option is to omit results that have already been widely reported, such as “networking within a limited group,” “stigma and violence compel MSM to remain hidden,” and “condom-less sex”. These could simply be mentioned in the Discussion. Instead, the focus should be placed on what the paper truly contributes, such as new emerging MSM who remain hidden. | We have reduced the results; by reducing quotations. As advice we have omitted some of the commonly reported themes. However the themes ‘condom less sex’ and ‘networking within a limited group’ has been renamed as ‘condom-less sex in hidden MSM’ and ‘limited group networking facilitates secrecy of the group’ respectively.  We are thankful to reviewers we have tried to bring the focus to new emerging MSM who remain hidden. | Results, page 11, line no. 211-212  Page 15, line no. 307 |
| 13 | ANSWERING THE QUESTION: DO THE DATA AND ANALYSES FULLY SUPPORT THE CLAIMS? IF NOT, WHAT OTHER EVIDENCE IS REQUIRED?  Elements that should be reviewed and improved in the Methods  According to the Methods section of your article, the manuscript “[reference 13]” defines that “All study participants were adults and of legal age.”  **Comment:** While the mean age is provided, given the special relevance of participants’ age—especially concerning the topic of sugar daddies or uncles—it would be highly relevant to include a table with the sociodemographic characteristics of participants, including: age, type of informant (key informant or population), region of origin, rural/urban setting, and other characteristics that are pertinent for interpreting the findings. | Thank you for the suggestion we have added the socio demographic tables for IDI, FGD and KII participants. | Page 65-67. Table 1 and 2 |
| 15 | The Methods section of your article also specifies: “MSM population categories recruited, and recruitment strategies are described in our previous report [reference 13].” However, in that study the dates and study sites differ (“four rural sites in Maharashtra, Odisha, Madhya Pradesh, and Uttar Pradesh between November 2018 and September 2019”).  **Comment:** It should be clarified whether this is the same study or a different one, given that this article includes more participants and more sites than the study mentioned in the Methods. If it is the same study, have the results of this article already been partially published before? | Thank you for pointing out we have clarified about the study states where rural and urban site were included. We have described all of them together now. Therefore now the study sites are matching with the earlier referenced paper. The timeline has also been clarified in the revised manuscript.  The results of this study have been partially published with addressing health seeking behaviour of rural MSM. Thank you for asking this question. We have mentioned this information in the revised manuscript. The current manuscript explores issues of new categories of MSM. | Materials and methods, page 7,  line no. 110-117, and 120-122  Discussion Page 42, line 901-903 |
| 16 | In the Methods section, it is stated: “Following purposive sampling techniques, 42 In-depth Interviews (IDIs), 40 Key Informant Interviews (KIIs), and 16 Focus Group Discussions (FGDs) leading to a total coverage among 98 participants, were conducted.”  **Comment:** It is not clear why both interviews and focus groups were conducted. | In-depth Interviews were conducted with hidden MSM who discuss their experiences and issues in a very confidential environment. FGD was conducted with visible MSM who acted as expert in the community issues, discussed the issues of hidden MSM and gave opinions on solutions for them. So the purposes were different and hence the study tools were different. This has been clarified in the revised manuscript. | Materials and methods,  Page 7-8, line no. 126-139 |
|  | Furthermore, of the 82 participants in interviews and the 16 FGDs, does the total of 98 participants include those who joined FGDs, or are they counted separately? It would be helpful to indicate how many individuals participated in FGDs and whether these were the same subjects who also participated in interviews. | The number of participants have been added in the revised manuscript. | Materials and methods, Page 7, line no. 122-125 |
| 17 | In the Methods section, the article specifies: “Each participant was given a unique identification number which included alphabet/s abbreviations to identify state, Urban (U) or Rural (R) setting and type of data collection tool used and the number.”  **Comment on identifiers:** While U and R are clear, the other elements (e.g., MH in [##MH-U-IDI-03##]) should also be explained. It would be important to show how the identifiers are constructed and how they relate to key aspects of the analysis.  Moreover, considering the declared study objective (“to understand the population which is hidden, their issues and their expectations…”), the number of interviews (82) is strikingly high for a qualitative study, particularly with hard-to-reach populations. Therefore, it is important to clarify: whose voice are we hearing? Information such as the age of the informant, place of residence, and—if available—the behavioral profile that led to their inclusion, would greatly strengthen the analysis. | As suggested the abbreviations in the ID numbers have been described in the revised manuscript.  We have also added other small demographic and behavioral profile only for 42 IDI in the revised manuscript. | Materials and methods, page 8,  Line no.144-148 |
| 18 | Additionally, conducting FGDs with highly stigmatized populations may itself contribute to stigma, as participants are required to share experiences in front of strangers. It would be important to explain: Why did you choose to conduct FGDs? How was confidentiality ensured for participants who, by definition, hide their identity for social and legal reasons? | The FGD were conducted among homogenous group of 'visible' MSM who volunteered to participate.  Each potential participant was counselled that no personal issues need to be discussed. Risk of breach of confidentiality was explained. Moderators were trained to divert discussion if any personal information was coming out inadvertently.  This information is added in revised manuscript. | Materials and methods, page 7-8,  Line no.131-139 |
| 19 | Finally, was there any case in which a sugar daddy or uncle declared having sexual relations with minors? If so, how was this handled? | No it was either in the key informant interviews or focused group discussions, information emerged regarding sugar daddies having sexual relations with minors. Debriefing was done after each interview.  This has been added in the revised manuscript. |  |
| 20 | According to the Methods section, it is also stated: “Emerging themes were identified based on the categories formed following repeated iterations using the grounded theory approach [14].”  Comment: It would be valuable to explain how the major codes of analysis were configured, and whether any categories emerged during the study that had not been previously anticipated. | The major codes of analysis were configured using grounded theory approach with initial 310 codes which got reduced to final categories. This has been explained in the revised manuscript.  New theme emerge as expanding demographics of hidden MSM which is included in the present manuscript. | Materials and methods, page 8-9, line no. 154-160  Results, page 10-11, line no.201-204 |
| 21 | The Results section presents findings by broad themes but not by specific groups (hijra/transgender, Panthis, Kothis, or by age groups). If there are findings relevant to these categories, they should be highlighted. | Thank you for a very relevant suggestion. We have added specific groups and age of those respondents who quotes have been included in the manuscript. This has strengthened our result. |  |
| 22 | Additionally, the first-time terms such as “chakka” or “kothi” appear, the meaning should be included in brackets. | Thank you for the suggestion, we have incorporated it in the study | Introduction, page 6, line no. 99  Materials and methods, page 8, line no. 136-137  Results, page 12,  line no. 242-243 |
| 23 | It is also recommended to include COREQ (Consolidated Criteria for Reporting Qualitative Research) standards in the methodology. | Thank you for the suggestion we have incorporated the COREQ guidelines in the methodology | Materials and methods, page 7-9, line no. 110-167 |
| **COMMENTS ON CONTENT** | | | |
| 24 | On the treatment of child abuse in the context of previous literature on sexual violence and child abuse  Comment: The article clearly describes the presence of sugar daddies or adult men (50 years or older) who offer money to young people or children in exchange for unprotected or non-consensual sex. My central concern is with the way the data are named and analyzed, where consensual sex and coercive/forced sex are treated as equivalent “barriers” to HIV prevention, as if these practices were comparable in behavioral terms.  While all these behaviors may indeed result in similar prevention challenges, the lack of a critical position in the text and the “neutral” treatment of these practices risk inadvertently invisibilizing criminal behavior and sexual violence against minors. | We sincerely thank our reviewer for this critical criticism. It has helped us in relooking at the analysis and discussion. We have revised the manuscript to bring a strong focus on sexual violence in child abuse.  We have removed the neutral treatment of these practices from the manuscript. Once again thank you. | Results, page no. 18-27, line no. 366-558  Discussion,  Page 40-48, line no. 871-1044 |
| 25 | International guidelines are clear: WHO, UNICEF, and UNAIDS recommend not using the terms “sexual debut” or “sexual initiation” when such events occur before the age of 18 through coercion, as this masks the violence. Instead, the recommended terms are sexual violence, child sexual abuse, or rape (when penetration and coercion occur). In HIV epidemiology, a distinction is explicitly made between consensual sexual debut and forced sexual debut, a category widely used in gender-based violence and sexual and reproductive health research. | We sincerely thank our reviewer for this critical comment and accordingly we have revised the manuscript to bring a distinction explicitly between consensual sexual debut and forced sexual debut. Once again thank you. | Abstract, page 3  Line no.43- 49,  Results, page 18, Box-1,  Page 19, line -386  Page 23, line no. 472-474  Discussion  Page 43, line no. 936-944,  Page 44, line no. 960-962 |
| 26 | The manuscript states: “In India, male child sexual abuse remains highly taboo, making acceptance and disclosure difficult.”  It appears that this taboo is reproduced, albeit unintentionally, in the manuscript, which does not explicitly denounce the violation of the human rights of society’s most vulnerable members—children. The text argues that adult men who sexually abuse children should be addressed through HIV prevention programs, while minors, victims of sexual assault, should be “empowered.” These statements should ideally be reviewed in light of human rights frameworks, child protection principles, and Indian law. | We again thank the reviewer for bringing clarification and correct strength to the manuscript. We have revised the manuscript in the light of human rights frameworks, child protection principles, and Indian law. | Discussion, page 51, line no. 1121-1129 |
| **SPECIFIC EXAMPLES** | | | |
| 27 | In the Abstract: “Both at urban and rural settings, the sexual debut of the child was between 2nd standard and 6th standard… The ‘sugar daddies/older MSM’ who were >50 years old seemed to slip from the continuum of programmatic prevention umbrella out of boredom, burn out, denial of self-risk, and the need to experiment with young boys.”  **Comment:** This phrasing should be reconsidered, as it risks “normalizing sexual violence against minors” as a “need.” Perhaps sugar daddies do not disengage from programs merely due to boredom or denial, but because they are abusing children. | We agree with the reviewers and we have revised the abstract accordingly to bring in the focus on evidence of sexual abuse of minors by older MSM. | Abstract, page 3, line no. 43-50 |
| 28 | In the Discussion: “The study shows an emergence of critical themes which summarises the experiences of the hidden and complex vulnerabilities of boys and men.”  **Comment:** By referring to “complex vulnerabilities of boys and men”, the text appears to equate the vulnerabilities of minors with those of 50-year-old adults. While both groups may experience vulnerability, children are not only vulnerable—they are being victimized by adults. The absence of explicit recognition could risk invisibilizing this, thereby exacerbating the vulnerability of abused children. | Thank you for the suggestion we have updated the statement. The study shows an emergence of critical themes which summarises the experiences of the hidden and complex vulnerabilities of all hidden MSM described above. The most concerning findings are the reported sexual abuse of boys and younger men by older MSM viz. Sugar daddies. | Discussion, page 43-44, line no. 937-946 |
| 29 | In the Discussion: “As reported in several studies from India and elsewhere, our study confirms a very young age of sex initiation (6–7 years), obviously condom-less. This early sexual debut (consensual/coercive/exploited) has been associated with increased risk of unintended pregnancy, STIs and future risky sexual behaviour.”  **Comment:** Placing “consensual, coercive, or exploited” in parentheses, separated by commas, makes them appear as variants of the same phenomenon, when in reality they are fundamentally different. Referring to rape or human sexual exploitation as “sexual debut” is problematic and should be revised. | We have revised as per the advice received from reviewers. The term sexual debut has been revised in context of adolescent boys to reflect sexual abuse. | Discussion, page 43, line no. 941-946 |
| 30 | In the Discussion: “The early initiation of adolescent MSM and their sexual interactions with sugar daddies underscores the urgent need to empower and educate young boys…”  **Comment:** Here again, the term “empower” is not appropriate. Children and adolescents must be protected by law, not “empowered or educated.” Empowerment implies restoring agency to a subject capable of exercising it, but a minor cannot exercise power in such circumstances. Therefore, the appropriate response is protection, not empowerment. | We agree with the criticism and we have brought in the focus towards protection and removing empowerment in case of children and adolescent in this study. | Discussion, Page 47,  Line no. 1028-1037 |
| 31 | Conclusions and Recommendations: The manuscript states that MSM-focused services with affirmative care, peer referral models, and mental health counseling could facilitate access among hidden MSM, and that “there is an urgent need to revisit strategies for engaging middle-aged MSM, the ‘sugar daddies’ or ‘uncles.’”  **Comment:** This treats sugar daddies merely as individuals in need of prevention or mental health programs, while overlooking the criminal nature of engaging in sexual relations with minors. | We have revised the conclusion and recommendation as per the suggestions of reviewers by bringing in the criminal nature of sugar daddies in engaging in sexual relations with minors | Conclusion,  Page no. 53-54, line no. 1172-1188 |
| **ON THE INTRODUCTION** | | | |
| 32 | The Introduction states: “The Indian National Strategic Plan for HIV/AIDS and STI (2017-24) focuses on Community Strengthening Strategy (CSS) with definite roles for KPs who are the centre of Community Strengthening activities of the HIV prevention and control program [11].”  Comment: It would be very valuable if the authors could critically articulate the Plan’s position on child sexual abuse and exploitation, and provide recommendations in this regard. | CSS does not address issues of sexual abuse and exploitation. We have removed this reference. |  |
| **FINAL RECOMMENDATION** | | | |
| 33 | If the paper is considered unsuitable for publication in its present form, the study nevertheless shows sufficient potential to merit revision and resubmission. It is recommended that the methodological aspects noted above be clarified, and that the manuscript be reframed through the lens of human rights, sexual violence, and child protection. This includes making visible the issue of sexual abuse and clearly distinguishing consensual sex from coerced or exploitative sex throughout the analysis. | The entire manuscript has been revised including the visible issue of sexual abuse coercive and exploitative sex. |  |
